# Chromosome 6q mediated loss of LATS1 tumour suppression as a targetable mechanism of ultra-high-risk posterior fossa A ependymoma

**DOI:** 10.1007/s00401-026-03098-z

**Published:** 2026-10-13

**Authors:** Andrew M. Donson, Andrea M. Griesinger, Rebecca J. Chapman, Richard G. Grundy, John-Paul Kilday, Vijay Ramaswamy, Jean M. Mulcahy Levy, Michael L. Kaufman, Vladimir Amani, Julia K. Sundheimer, Kristian W. Pajtler, Nicholas K. Foreman, Timothy A. Ritzmann

**Affiliations:** 1 Morgan Adams Foundation Pediatric Brain Tumor Research Program, Children’s Hospital Colorado, Aurora, CO 80045 USA; 2 Department of Pediatrics, University of Colorado Denver, Aurora, CO 80045 USA; 3 Children’s Brain Tumour Research Centre, School of Medicine, University of Nottingham, Nottingham, UK; 4 The Geoffrey Jefferson Brain Research Centre, University of Manchester, Manchester Academic Health Science Centre, Manchester, UK; 5 Children’s Brain Tumour Research Network (CBTRN), Royal Manchester Children’s Hospital, Manchester University NHS Foundation Trust, Oxford Road, Manchester, UK; 6 Neuro Oncology Program, Hospital for Sick Children, Toronto, ON Canada; 7 Department of Biomedical Informatics, University of Colorado Anschutz Medical Campus, Aurora, CO 80045 USA; 8 University of Colorado Cancer Center, University of Colorado Anschutz Medical Campus, Aurora, CO 80045 USA; 9 Hopp Children’s Cancer Center at the NCT Heidelberg (KiTZ), 69120 Heidelberg, Germany; 10 Division of Pediatric Neurooncology, German Cancer Research Center (DKFZ), German Cancer Consortium (DKTK), Im Neuenheimer Feld 580, 69120 Heidelberg, Germany; 11 Department of Pediatric Hematology, Oncology and Immunology, Heidelberg University Hospital, Im Neuenheimer Feld 430, 69120 Heidelberg, Germany

**Keywords:** PFA, Ependymoma, Chromosome instability, LATS1, Hippo signalling

## Abstract

Posterior Fossa A (PFA) is the most frequent and clinically aggressive ependymoma (EPN) subgroup, with relapse typically representing a terminal event despite intensive multimodal therapy. Ultra-high-risk PFA EPNs are characterised by chromosome 6q loss (6q-) with or without chromosome 1q gain (1q +). While the molecular mechanisms driving 1q+ have been elucidated, 6q- PFA pathobiology remains poorly understood. Here, we show that loss of 6q in PFA EPN results in deletion of *LATS1* (Large Tumour Suppressor Kinase 1), thereby dysregulating the Hippo signalling pathway and enhancing YAP/TEAD activity. Integrated analyses of primary and recurrent PFA EPNs, incorporating clinical, multiomic, and functional assays, were performed to define the biology and therapeutic susceptibility of 6q- PFA EPNs. Cumulative 6q loss was associated with nearly half of PFA EPN-related deaths. Copy number profiling identified the *LATS1* locus as recurrently deleted across all 6q- cases, including 10/60 with partial 6q deletions. Restoration of *LATS1* expression in 6q- PFA cell lines re-established protein levels and suppressed proliferation. Tumour analyses showed marked Hippo pathway disruption with elevated YAP expression and *TEAD2-*related transcriptional activity. TEAD2 palmitoylation inhibitor VT107, and Proteolysis Targeted Chimera (PROTAC) degrader H122, exhibited PFA 6q- selective activity in vitro. *TEAD2* upregulation was further observed in a previously described undifferentiated, progenitor-like population of PFA EPN cells. In conclusion, 6q loss defines an aggressive biological subset of PFA associated with dismal outcomes. Identification of *LATS1* as a recurrently lost tumour suppressor implicates Hippo pathway dysregulation as a central driver of malignancy, and we identify YAP/TEAD signalling as a potential therapeutic target.

**Supplementary Information:**

The online version contains supplementary material available at https://doi.org/10.1007/s00401-026-03098-z.

## Introduction

Ependymoma (EPN) is the second most common malignant brain tumour in childhood and comprises multiple molecular entities [32, 33]. Posterior fossa A (PFA) EPN is the most frequent subgroup, affects the youngest children, and carries the poorest outcomes [32, 40]. Despite modern therapy targeting maximal tumour resection followed by focal radiotherapy [42], relapse occurs in around 50% of cases, and only 25% of these children survive beyond 5 years [40, 56]. PFA EPNs showing chromosomal instability—typically gain of chromosome 1q (1q +) and/or loss of chromosome 6q (6q-) exhibit an especially dismal prognosis, forming an ultra-high-risk group for whom current standard-of-care interventions are rarely curative [5].

We previously hypothesised that certain molecularly defined EPN subgroups are destined to have poor outcomes [39]. Since then, we have presented evidence from a large, international cohort showing that 1q+ and 6q- increase in frequency during PFA EPN recurrence and progression. Up to two-thirds of patients acquire a high-risk copy number alteration by first relapse, compared to one-fifth at initial presentation [10]. This evolution of tumour biology may explain why recurrence is associated with such dismal outcomes.

Recent work into the biology of 1q+ in PFA progression has highlighted the role of *MDM4,* located on chromosome 1q, as a negative downstream regulator of P53, mimicking a P53 mutation [15]. In addition, *UCK2,* also on 1q, represents a potential therapeutic weakness that may render 1q+ PFA EPN sensitive to the chemotherapy agent 5-Fluorouracil (5-FU). This hypothesised therapeutic intervention is now a focus for the development of biology-driven clinical trials of 5-FU and radiotherapy in both North America (ACNS2422) and Europe.

In contrast, the biological basis for the poor prognosis associated with 6q- PFA EPN remains undefined. Here, we investigate the tumour biology of 6q- PFA EPN using an established international dataset. We hypothesise that the adverse outcome associated with 6q- results from the loss of the tumour-suppressor gene *LATS1*, leading to dysregulation of the Hippo pathway and offering a druggable target through inhibition of YAP/TEAD signalling.

## Methods

### Cohort details

Matched primary and recurrent tumour sample copy-number variation (CNV) data was previously obtained by DNA methylation profiling in our prior multi-institutional study [10]. This dataset included 101 children with recurrent, DNA methylation-classified PFA EPNs.

For additional experiments beyond the original multi-institutional cohort, immunohistochemistry was performed on archival FFPE material from 75 PFA EPNs (25 6q- and 50 wild type), which were obtained from Children’s Colorado Hospital. Xenium in situ spatial transcriptomics was performed on 14 snap-frozen tumour samples from the Colorado paediatric neuro-oncology brain tumour bank. Bulk RNAseq, Immunohistochemistry and Xenium samples were obtained during routine surgery or autopsy respectively, with institutional approval and patient consent (COMIRB 95–500).

### Bulk RNA sequencing analysis

Bulk RNA-seq analysis was performed on a dataset of primary and recurrent PFA (GSE226961, GSE243682, GSE327676)[10, 13]. RNA was isolated from snap-frozen material and sequenced on the Illumina NovaSeq 6000 platform. Sequencing data were aligned using STAR [8], and transcript quantification (in fragments per kilobase per million reads, FPKM) was generated with Cufflinks [50]. Single-sample gene-set enrichment analysis (ssGSEA) was performed on individual RNA-seq FPKM data using the GenePattern portal [4, 45].

### Survival analysis

Univariate analyses using the Kaplan–Meier method were employed to assess the effect of whole or partial loss of chromosome 6q on survival from both primary disease and recurrence. We also evaluated the impact of gene expression and transcription-factor target gene enrichment score (ssGSEA) on survival using the same method. Progression free survival was modelled as a time-dependent variable to capture the effect of CNV changes and associated biological effects at recurrence. This approach addresses the significant outcome impact of 1q gains and/or 6q losses at recurrence in PFA, which significantly confound overall survival analyses.

### LATS1 knock-in experiments

LATS1 open reading frame (ORF) knock-in expression vector was designed and purchased through GeneCopia. Lentiviral LATS1 vector (hLATS1) and corresponding control vector (Ctl) were generated using the pLKO system, where LATS1 and control vectors are transfected into HEK293FT cells with psPAX and pMD2.G viral packaging plasmids and TransIT-LT1 Reagent (Mirus Bio). For in vitro assays, viral supernatant was collected in Opti-MEM with 15% FBS and 1% pen/strep. LATS1 ORF knock-in was performed in two PFA 6q- cell lines previously established and characterized in our laboratory (MAF-811; MAF-928)[1]. We used three comparator cell lines with PFA 6q WT: two supratentorial EPN ZFTA-fused cell lines (MAF-1329 [19] and DFKZ-EP1NS [30]) and a paediatric glioblastoma (GBM) cell line SF188 [9]. All cell lines were subjected to routine authentication by DNA fingerprinting and mycoplasma testing. For this study cell lines were propagated under standard tissue culture conditions and grown in fetal bovine serum (FBS)-supplemented media as monolayers, Cells used in in vitro experiments were transduced with viral supernatant using 8 μg/mL polybrene. LATS1 knock-in was confirmed by Western blot analysis.

### In vitro comparative TEAD palmitoylation inhibitor VT107 and TEAD PROTAC degrader H122 drug screen

We screened the effect of TEAD palmitoylation inhibitor VT107 (MedChemExpress. Cat. no. HY-134957) [48] and TEAD PROTAC (proteolysis-targeting chimera) degrader H122 (MedChemExpress. Cat. no. HY-172113) [27] in PFA 6q- cell lines MAF-811 and MAF-928 and a panel of comparator paediatric brain tumour cell lines as previously described to identify ependymoma selective drug sensitivity [9]. These included EPN PFA 6q WT cell line DKFZ-BT232 [47], EPN ZFTA-fused cell lines MAF-1329 and DFKZ-EP1NS and non-EPN paediatric high-grade glioma (HGG) cell lines (diffuse intrinsic pontine gliomas DIPG-IV and glioblastoma SF188) and medulloblastoma (MED) cell lines (DAOY, ONS76, FH-411and D458) that were maintained as described previously [9]. Cell lines were propagated as monolayers apart from semi-adherent suspension cell lines FH-411 and D458.

Cell lines were plated at ~50% confluence in 96-well plates and treated with a range of doses of VT107 (range = 10μM to 0.6 nM) and H122 (range = 1000 nM to 0.06 nM) for 72 h. Cell proliferation was measured using tritiated thymidine incorporation as previously described [9]. Treatment dose curves and associated IC-50 values were modelled using Prism statistical software (GraphPad Software, Inc., La Jolla, CA).

H122 PROTAC-specific degradation of TEAD was determined by Western blot analysis of protein extracted from PFA 6q- cell lines treated with a range of H122 doses (1000 nM to 8 nM) for 4 h. Determination of the baseline endogenous TEAD degradation rate in PFA 6q- cell lines was performed by extracting protein at 4 h and 24 h after treatment with protein translation inhibitor cycloheximide (100μγ/ml; Sigma Cat. no. C-6679).

### Western blot

Western blots were performed as previously described [52]. Primary antibodies used were anti-LATS1 (1:1000, Proteintech #17,049–1-AP), anti-phospho-YAP (S127) (Cell Signaling, #4911), pan-TEAD (Cell-signaling #13,295), DNMT1 (Cell Signaling #5032) and anti-β-actin. Densitometry was performed using ImageJ software.

### YAP immunohistochemistry

Standard immunohistochemistry was performed for YAP (1:200, antigen, Santa Cruz sc-101199) with antigen retrieval (10 mM Sodium Citrate pH 6.0, 110 °C, 10 min) and counterstained with hematoxylin, using techniques described previously [13]. Blinded scoring was performed using a subjective scoring scale (range 0–4) (AMD).

### Xenium *in-situ* spatial transcriptomic analyses

The spatial expression of TEAD2 in PFA 6q- (n=5) and PFA WT (n=9) neoplastic subpopulations was performed using Xenium in situ spatial transcriptomics (10X Genomics). Snap-frozen samples were embedded in Optimal Cutting Temperature (OCT) compound, cryosectioned at 10μM thickness (Leica CM1520 cryostat) and mounted onto Xenium Slides. Slides were processed on a Xenium Analyzer (Instrument software v3.3.0.0.; analysis v 3.3.0.1) following the manufacturer’s instructions. The Xenium Analyzer detected and amplified RNA, DNA, and protein probes in 15 serial decoding and imaging cycles whilst maintaining tissue integrity and producing images of whole tissue sections with single-cell and single-molecule resolution. A custom gene probe panel of 480 genes was synthesized that included marker genes of previously identified cellular subpopulations in ependymoma [13–15] and other paediatric brain tumour diagnoses [7, 37, 57] (Supplementary Table 1). RNA probes were hybridized overnight, ligated, and then amplified using rolling circle amplification according to the manufacturer’s instructions (10× Genomics CG000578, CG000580, CG000613). All incubations were performed using reagents provided by 10× Genomics on an XT96 PCR machine (VWR). Autofluorescence was then quenched and morphology stains added for cell segmentation, either nuclear (DAPI) (samples 812–1, 821–1, 928–1, 723–1 and 723–2) or, cytoplasmic (18S RNA, alphaSMA and vimentin protein), and membrane (ATP1A1, CD45, E-cadherin) stain cell segmentation cocktail for improved cell segmentation (samples 928–2, 897–1, 897-X, 987–1, 911–1, 1536–1, 1536–2, 459–1 and 459–2). Slides were then loaded onto the Xenium Analyser for whole-slide tissue overview scanning and region selection. Tissue regions were denoted, and the onboard cyclic decoding and imaging occurred over a 20 – 48 h period. Data were analysed on the instrument after image acquisition, performing probe deconvolution, cell segmentation, and probe graph-based clustering. Quality control, image heatmaps and run data were reviewed in the Xenium Explorer (4.1.0) software. After Xenium analysis, H&E staining was performed on Xenium slides and whole-slide scans were performed (PhenoImager HT: 20× objective, 0.5 micron resolution). Images were converted to OME. TIFFs file using QuPath v0.5.1 and imported to Xenium Explorer and aligned with tissue images.

Cell cluster identities were assigned based on overlap with gene expression signatures from PFA neoplastic and non-neoplastic subpopulations identified by single-cell and single-nuclei RNAseq [10, 14] and Visium spatial transcriptomics [13] using hypergeometric analysis. For each sample, neoplastic clusters were then consolidated into five categories (ciliated (CEC), transportive (TEC), mesenchymal (MEC), undifferentiated-A (UEC) and proliferative undifferentiated-A (UECprolif.) EPN cells. Per cell TEAD2 and LATS1 transcript counts for each cluster were then extracted for comparative inter-sample and inter-subpopulation analyses. Xenium spatial transcriptomic data is available at GEO (GSE327676).

## Statistics

Statistical analyses were performed using R, GraphPad Prism, and Excel (Microsoft) software. Kaplan–Meier survival curves were compared with log-rank tests, and p<0.05 was considered statistically significant unless otherwise stated.

## Results

### Cumulative loss of chromosome 6q underlies almost 50% of PFA deaths

**Fig. 1:**
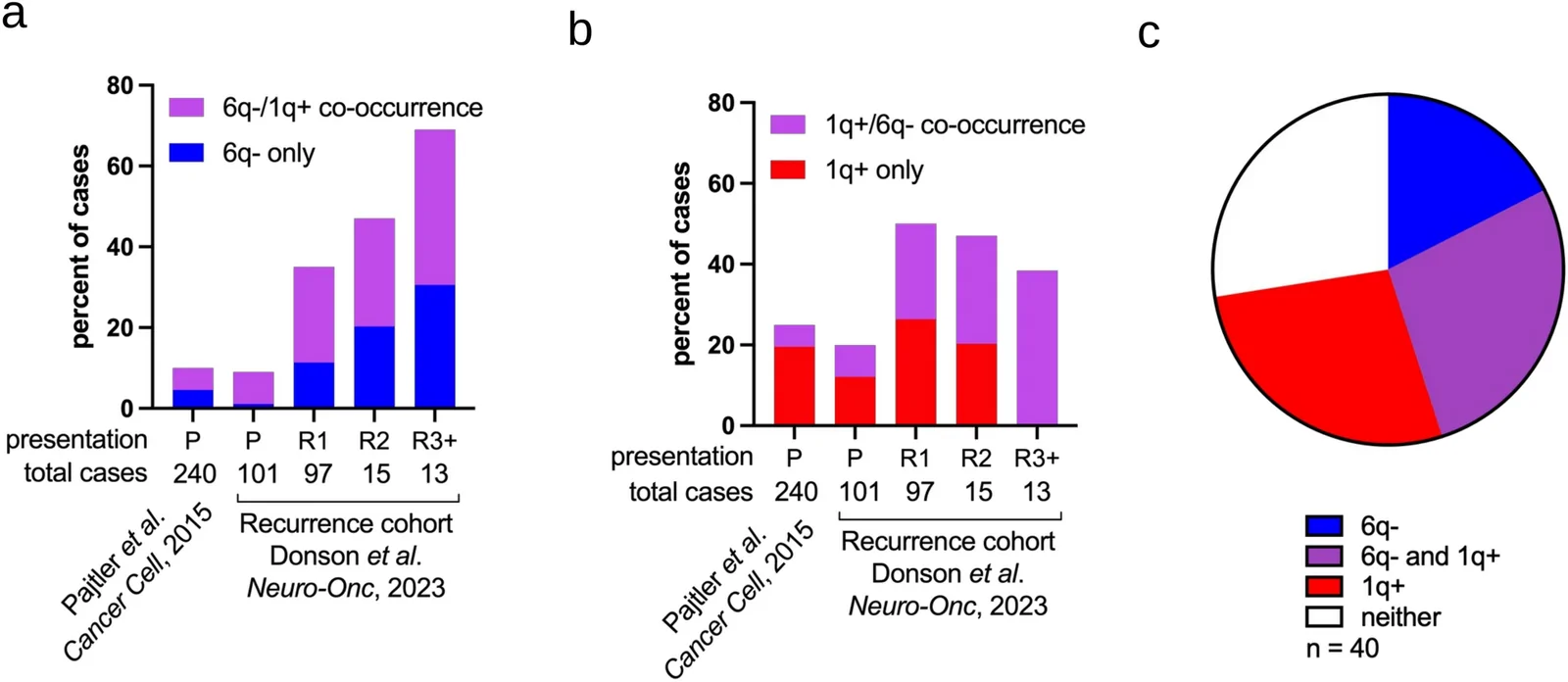
Impact of 6q loss (6q-) in late recurrence and death in PFA. Cumulative acquisition of **a** 6q- and **b** 1q+ in successive PFA recurrences. **c** Proportion of acquired 6q-, 1q+ or 6q-/1q+ co-occurrence in PFA tumours at time of death. Abbr: P, presentation: R1; 1st recurrence; R2, 2nd recurrence; R3+, 3rd and subsequent recurrence and autopsy combined

We re-analysed data from our previously published international cohort of primary and recurrent ependymomas [10] to provide a more detailed view of the high-risk copy-number changes, focusing on chromosome 6q- (Fig. 1). Compared with 1q+, 6q- was relatively uncommon at first diagnosis, occurring in 4% (3/75) of tumours in isolation, and 8% (6/75) when co-occurring with 1q+. The distribution was consistent with data from a large independent cohort [33] (Fig. 1a). The proportion of tumours with 6q- increased progressively across recurrences, affecting 34% (25/73) at the first recurrence, 47% (8/17) of the cohort by the second recurrence, and 69% (9/13) of the third and later recurrences. Both isolated 6q- and 1q+/6q- co-occurrence rose over time (Fig. 1a). In contrast, 1q+ appeared early – affecting 48% (36/75) of cases at first recurrence and then remained relatively stable, with 100% (5/5) of 1q+ co-occurring with 6q- by third and later recurrences (Fig. 1b).

Consistent with prior studies delineating the high-risk behaviour of 1q+ in EPN [2, 5, 25, 29, 38], patients with 1q+, either alone or combined with 6q-, accounted for 55% (22/40) of all deaths. However, 6q- was also strongly associated with fatal outcomes; tumours harbouring 6q- (either alone or co-occurring with 1q +) also represented 45% (18/40) of all deaths, underscoring its major contribution to mortality in this cohort (Fig. 1c).

### The *LATS1* locus is lost in both whole and partial 6q- ependymoma, and outcome is poor irrespective of extent of loss

**Fig. 2:**
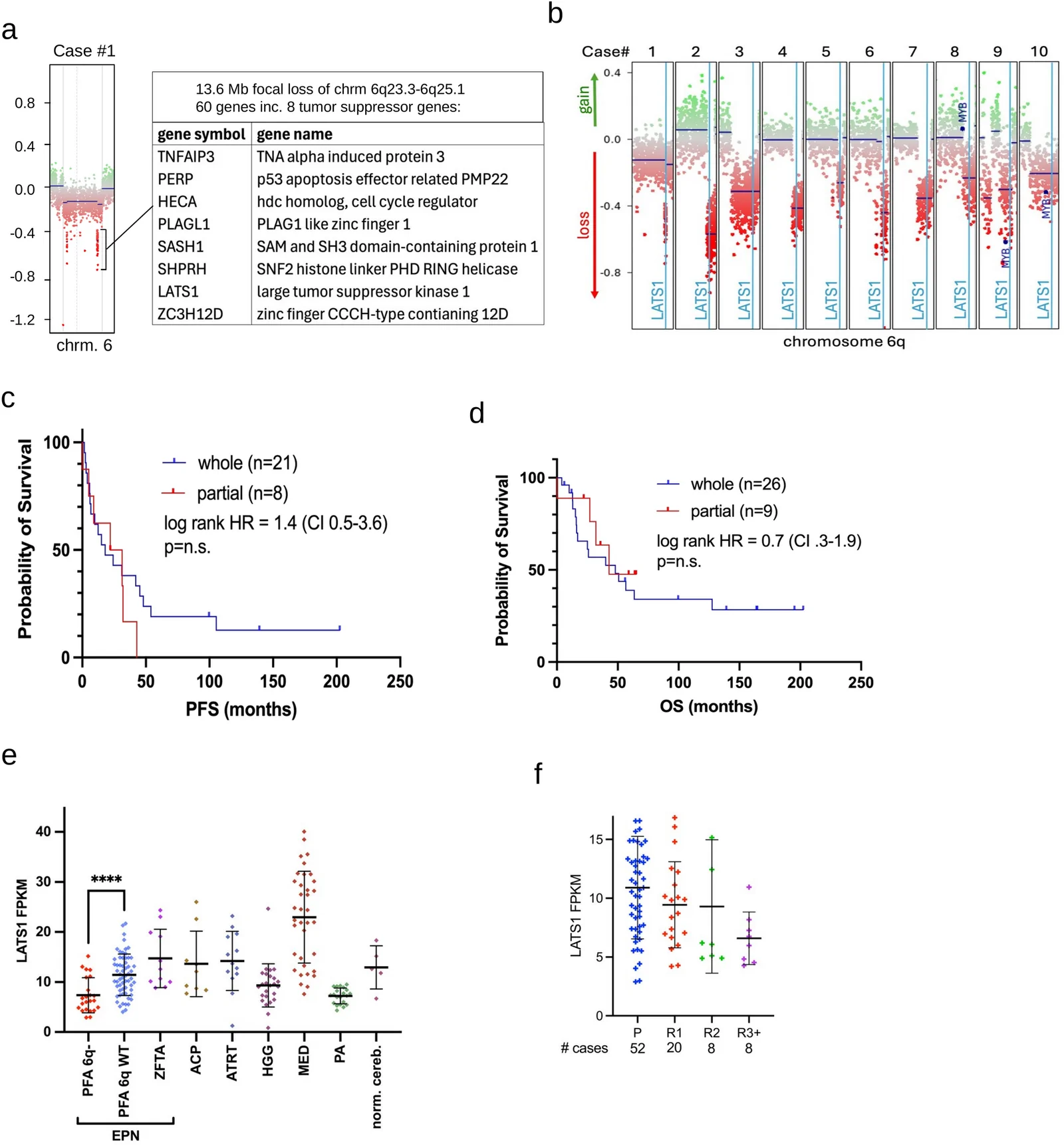
Candidate 6q tumour suppressor genes in the aggressive PFA 6q- phenotype. **a** PFA autopsy sample (case #1) showing a 60 Mb focal loss in 6q-, spanning 60 genes, including 8 tumour suppressor genes. **b** 10 of 60 PFA 6q- cases examined had partial loss of 6q all of which encompassed the LATS1 locus. **c** PFS and **d** OS for partial loss versus complete loss of 6q in PFA. **e** LATS1 transcript in PFA 6q- (n=23), PFA 6q WT (n=60), a panel of other paediatric brain tumour types and normal cerebellum (ACP, adamantinomatous craniopharyngioma (n=9); ATRT, atypical teratoid rhabdoid tumour (n=13); HGG, high-grade glioma (n=25); MED, medulloblastoma (n=36); PA, pilocytic astrocytoma (n=21); normal cerebellum (n=5)). (f) LATS1 transcript expression decrease in successive recurrences in PFA (P=presentation; R1 = 1st recurrence; R2 = 2nd recurrence; R3+= 3rd and successive recurrences)

To explore the aggressive phenotype of 6q- PFA EPN, we sought to identify the gene or genes driving the 6q- phenotype. We first examined an autopsy sample (case−1), which showed a focal region of 6q loss spanning q23.3-q25.1, covering 13.6 megabases (Fig. 2a). Among 60 genes in this region, eight were documented tumour suppressors [55]: *TNFAIP3*, *PERP, HECA*, *PLAGL1*, *SASH1*, *SHPRH*, *LATS1*, and *ZC3H12D*. Of these, *LATS1* and *PLAGL1* have been previously linked to EPN. Large Tumour Suppressor Kinase 1 (*LATS1*) is an established tumour suppressor in a broad range of human malignancies [55] and, of the eight PFA 6q- minimal region of loss tumour suppressors, has the highest incidence of loss (1.5%) in a pan cancer study of tumour drivers [3]

This PFA 6q- focal region of loss also contained tumour suppressor *PLAGL1*, however *PLAGL1* has been reported as an oncogene in the context of EPN, therefore not consistent with the proposed loss of tumour suppressor function via 6q-. Specifically, *PLAGL1* fusions, resulting in high expression of *PLAGL1* transcripts, have been identified in a novel subset of paediatric supratentorial neuro-epithelial tumours, of which a high proportion were histopathologically diagnosed as EPN [44]. Additionally, a more recent study has shown that increased *PLAGL1* transcriptional activity is a result of ZFTA fusion in a subset of supratentorial EPN [24].

To understand the importance of this region, we reviewed the DNA-methylation-derived copy-number plots for all cases of 6q- in our cohort. Of 60 cases with 6q-, 10 (16.7%) demonstrated only partial deletions. Notably, all these areas of partial deletion encompassed the focal region of 6q loss as seen in case−1 that includes that *LATS1* locus (Fig. 2b), implicating its role as the principal gene associated with the PFA 6q- phenotype in those cases with partial 6q loss.

We next compared survival between tumours with partial versus whole 6q loss. Progression-free survival from the time of 6q- acquisition showed no significant difference between these two groups (log-rank HR = 1.6, 95% CI 0.4–4.5; 26.65 months for partial loss and 18 months for whole loss; Fig. 2c). Both exhibited poor overall survival, with median overall survival of around 50 months in either group (Fig. 2d).

Analysis of bulk RNA-seq data confirmed markedly reduced *LATS1* transcript expression in 6q- (n=18) compared with 6q wild-type (n=58) PFA EPN (Fold change = 1.5; 2-tailed p=2×10^–5^), *LATS1* in 6q- PFA EPN also had the lowest expression of any brain tumour type examined (Fig. 2e). Mirroring the increased 6q loss seen in successive recurrences of PFA (Fig. 1a) we observed a decrease in the expression of LATS1 transcript in successive recurrences (Fig. 2f). Unlike 6q loss however, LATS1 transcript expression was not associated with progression free survival in our bulk RNAseq cohort where samples were dichotomized to higher or lower than median *LATS1* expression for progression free survival (PFS), either in all cases (n=88), or after exclusion of cases with confounding high-risk clinical variables (1q gain, 6q loss, infants under 1 year old)(n=40).

### LATS1 knock-in restores protein expression and suppresses proliferation in 6q- PFA cell lines

**Fig. 3:**
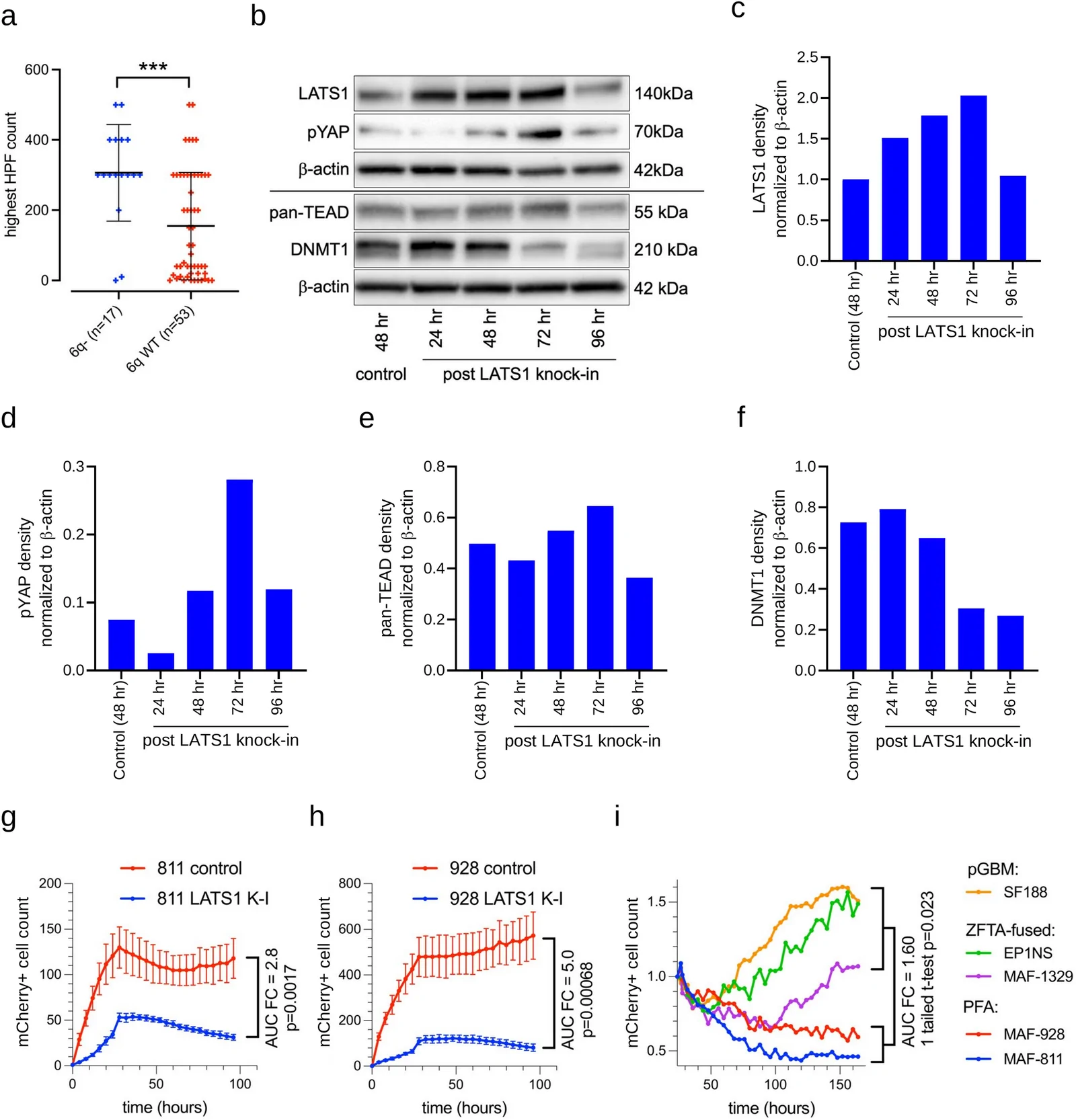
Impact of LATS1 loss on PFA 6q- proliferation. **a** Ki-67 positive cell count per highest high power field (HPF) in individual patient samples grouped according to (i) 6q- and (ii) 6q WT (*** p<0.0001; Student’s t-test, 2-tailed). **b** LATS1 lentiviral knock-in increases protein expression of (i) LATS1 and (ii) phospho-YAP (pYAP)(S127), (iii) pan-TEAD and (iv) DNMT1 in PFA 6q- cell line MAF-811, and associated densitometric quantification of **c** LATS1 protein knock-in, **d** pYAP, **e** pan-TEAD, and (f) DNMT1. LATS1 lentiviral knock-in blocks proliferation in PFA 6q- cell lines **g** MAF-811 and **h** MAF-928 compared to empty vector controls. (i) LATS1 knock-in blocks growth in PFA 6q- compared to 6q WT ZFTA-fused EPN and pGBM cell lines (Student’s t-tests, 1-tailed) as measured by mCherry positive cell count

To identify differences in cellular proliferation between 6q- and 6q wild-type PFA EPN, we reanalysed quantified Ki-67 proliferation indices by highest high-power field count from our prior study [10]. The 6q- tumours exhibited significantly increased Ki-67 levels (6q- n=17, 6q WT n=36, 2-tailed p = 0.0005), supporting the hypothesis that the 6q- PFA EPN cells have an intrinsically more proliferative phenotype (Fig. 3a).

Given the association between 6q-, increased Ki-67 indices, and poor prognosis [5], we investigated whether loss of *LATS1* contributes functionally to tumour growth. We performed *LATS1* lentiviral knock-in experiments and quantified LATS1 protein expression by western blot, normalised to beta-actin. In the 6q- PFA EPN cell line, MAF-811, LATS1 protein increased significantly at 24 h post-knock-in and continued rising through 72 h (Figs. 3b and c), remaining persistently above control levels.

Furthermore, *LATS1* restoration resulted in markedly reduced proliferation in two independent 6q- PFA EPN lines (MAF-811 and MAF-928), compared to controls (Figs. 3g,h). Moreover, compared with other established paediatric brain tumour lines (ZFTA-fused EPNs EP1NS and MAF-1329, and paediatric GBM line SF188), *LATS1-*reconstituted 6q- EPN cells displayed the most restricted growth patterns with a rapid fall in cellular proliferation, whilst the 6q wildtype lines maintained steady proliferation (Fig. 3i).

### Loss of chromosome 6q suppresses *LATS1* and activates the Hippo pathway

*LATS1* is a key negative regulator of the Hippo signalling pathway originally implicated in controlling organ size, cell fate, and proliferation, through S127 phosphorylation and subsequent degradation of YAP in the cytoplasm, preventing its transit to the nucleus where it binds and activates TEAD transcription factors [18, 28]. It acts as a tumour suppressor in multiple malignancies, including lung, breast, gastric, hepatic, renal, colorectal, and haematologic cancers [17, 34]**.**

Hippo dysregulation exists in several other EPN variants. A rare supratentorial EPN of childhood is driven by a Hippo pathway dysregulating YAP1-fusion [33]. NF2 mutant spinal ependymomas show Hippo dysregulation through the disrupted interaction of NF2 and LATS1 [36], and similarly, loss of chromosome 22 that includes the NF2 locus potentially disrupts LATS1 activity [23, 31, 36]. In addition to the formation of EPN-like tumours following LATS1 knockdown in neural progenitors in vivo, Eder et al. also showed YAP1 activation in this model produced EPN-like tumours [11]. We therefore hypothesised that *LATS1* loss in PFA 6q- deregulates Hippo signalling via release of YAP/TEAD activity and promotes their nuclear translocation.

**Fig. 4:**
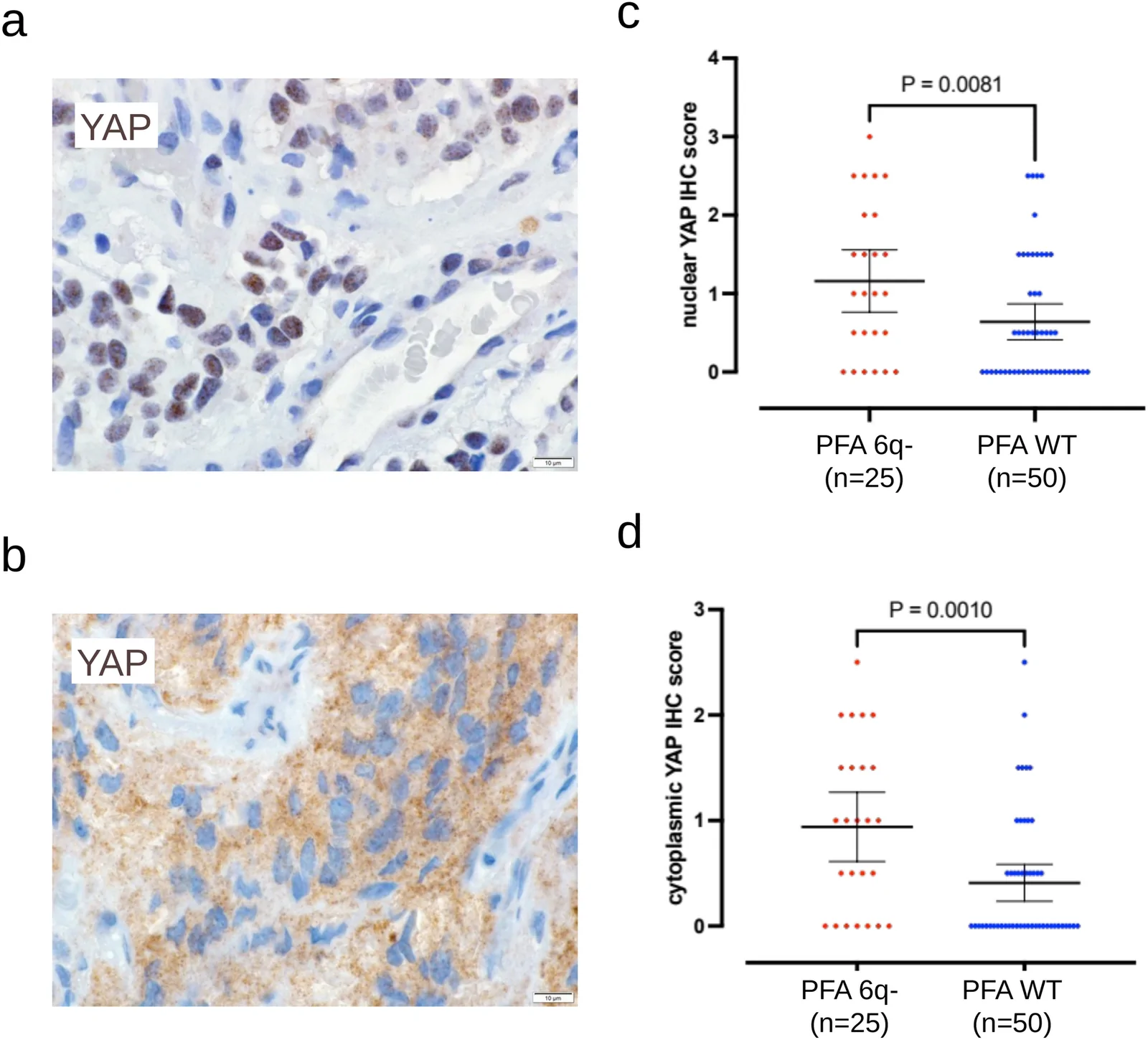
Impact of LATS1 loss on YAP expression in situ. Representative **a** nuclear and **b** cytoplasmic YAP protein expression in PFA 6q- **c** Nuclear and **d** cytoplasmic YAP score in a cohort of 75 PFA EPN

To test this hypothesis, we performed immunohistochemistry for YAP expression on a cohort of 75 PFA EPNs (25 6q- and 50 wild type). Both nuclear and cytoplasmic YAP expression were significantly higher in 6q- tumours (p = 0.0081 and p = 0.0010, respectively; Fig. 4), consistent with Hippo pathway activation in those cases with reduced LATS1. We further hypothesise that increased nuclear YAP drives TEAD expression, whereas elevated cytoplasmic YAP likely reflects reduced LATS1-mediated degradation. The functional impact of LATS1 on the Hippo pathway in PFA is indicated by increased phospho-YAP (S127) upon LATS1 knock-in in PFA 6q- cell line MAF-811 (Fig. 3b,d).

### TEAD2 expression is upregulated in 6q- PFA EPN and predicts poor survival

**Fig. 5:**
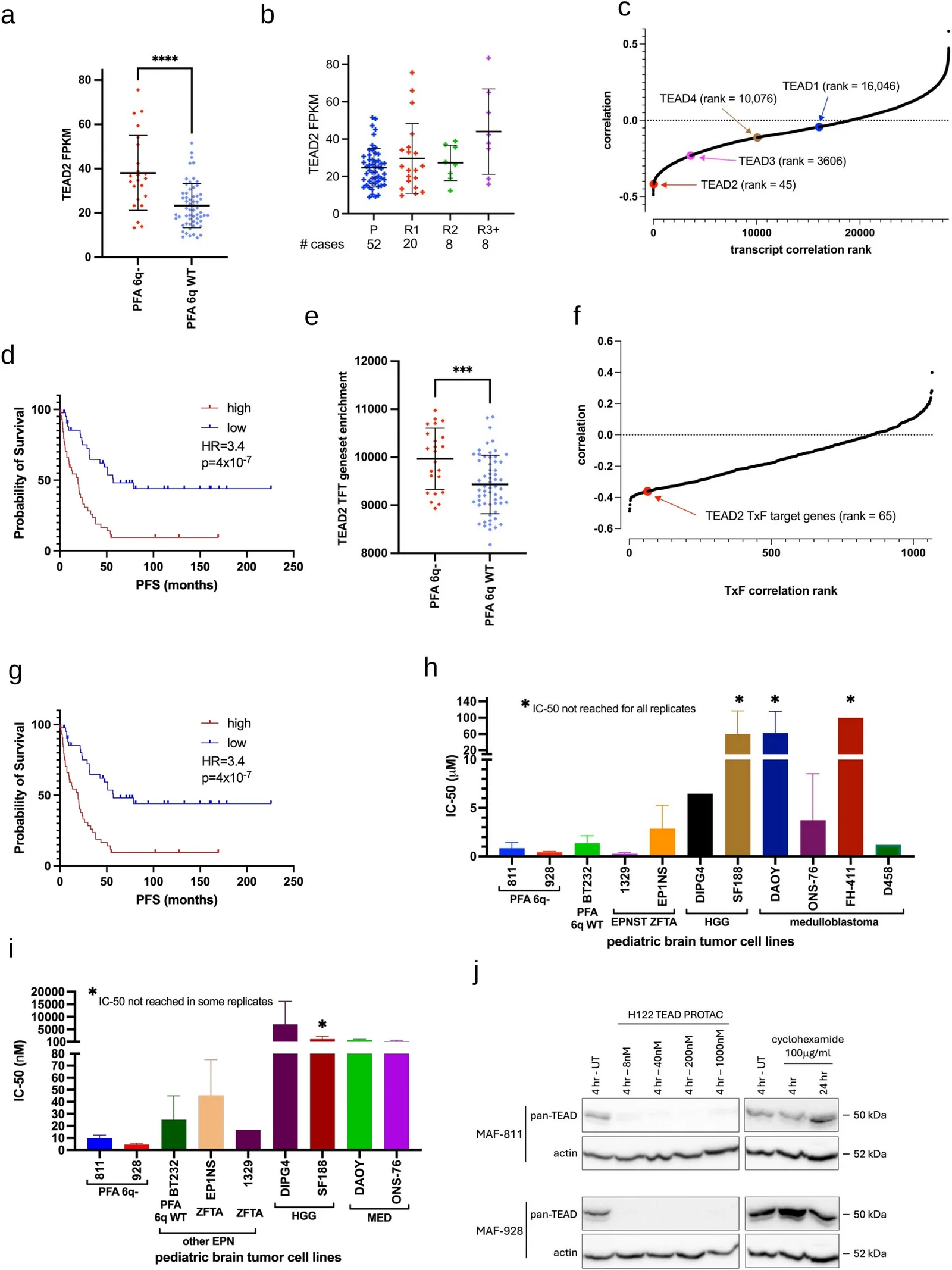
TEAD expression, prognostic impact and growth dependency in PFA 6q-. **a** TEAD2 transcript in PFA 6q- versus PFA 6q WT (**** p<10^–5^; Student’s t-test, 2-tailed). **b** LATS1 transcript expression decrease in successive recurrences in PFA (P=presentation; R1 = 1st recurrence; R2 = 2nd recurrence; R3+= 3rd and successive recurrences).**c** TEAD transcript ranking in 28,000 transcripts correlated with time to progression (TTP) in a cohort of 79 recurrent PFA samples from any presentation. **d** Progression free survival (PFS) from any presentation for higher than median TEAD2 transcript expression in PFA from any presentation (n = 88). **e** TEAD2 transcription factor target geneset enrichment (ssGSEA) in PFA 6q- versus PFA 6q WT (*** p<10^–3^; Student’s t-test, 2-tailed). **f** TEAD transcription factor targets (TFT) geneset enrichment (ssGSEA) ranking in 1066 transcripts correlated with time to progression (TTP) in a cohort of 79 recurrent PFA samples from any presentation. **g** Progression free survival (PFS) from any presentation for higher than median TEAD2 TFT geneset enrichment in PFA from any presentation (n = 88). **h** In vitro effect (IC-50 dose) of TEAD palmitoylation inhibitor VT107 on ^3^H-thymidine incorporation in PFA 6q- cell lines MAF-811 and MAF-928 and a panel of comparator pediatric brain tumor cell lines. **i** In vitro effect (IC-50 dose) of TEAD PROTAC H122 in PFA 6q- cell lines MAF-811 and MAF-928 and a panel of comparator pediatric brain tumor cell lines as measured by ^3^H-thymidine incorporation. (j) H122-mediated degradation of TEADs in PFA 6q- cell lines MAF-811 and MAF-928 at 4 h post-treatment at a range of concentrations (left). No significant endogenous degradation of TEADs was observed up to 24 h after inhibition of protein synthesis (100 mg/ml cycloheximide treatment) (right)

We next examined the involvement of TEAD transcription factors, the primary effectors of the Hippo signalling cascade, in PFA 6q-. Reanalysis of bulk RNA-seq data revealed significantly higher *TEAD2* RNA expression in 6q- (n=23) versus 6q wild-type (n=60) PFA EPNs (FC=1.63; p=2.08×10^–6^)(Fig. 5a). Consistent with this negative correlation between 6q- and increased TEAD2 RNA, we observed increased TEAD2 expression in successive PFA recurrence (Fig. 5b) whereas 6q is increasingly lost (Fig. 1a). TEAD4 was also significantly higher in PFA 6q- (FC=1.47; p=0.035), whereas TEAD1 and TEAD3 were not differentially expressed. Functionally, LATS1 knock-in did not significantly change pan-TEAD protein expression (Fig. 3b,e), consistent with TEADs role as a transcription factor in the Hippo pathway that is activated once bound to nuclear YAP.

To determine the relative prognostic association of TEAD1-4 gene expression, we correlated all transcripts’ gene expression values with time to progression (TTP) in 79 recurrent PFA from any presentation. *TEAD2* ranked 45th out of a total of 28,595 transcripts negatively correlated with time to progression (Fig. 5c). Higher than median *TEAD2* target-gene expression was associated with poor survival (HR = 3.2; p = 4×10^–7^)(Fig. 5d). Similar trends were observed for *TEAD3* (HR = 1.8; p = 0.017) and *TEAD4* (HR = 1.9; p = 0.013), where high expression correlated with worse outcomes.

The Molecular Signatures Database (MSigDB; UC San Diego and Broad Institute) includes Transcription Factor Target (TFT) genesets (n=1066) of which TEAD2 is included, but not TEADs 1, 3 or 4. *TEAD2* TFT single sample gene set enrichment (ssGSE) scores were higher in 6q- cases (n=23) versus 6q wild-type (n=60) PFA EPNs (p = 3.44×10^–4^)(Fig. 5e). The most significantly overexpressed TEAD2 TFTs in PFA 6q- cases included DNMT1 (DNA methyltransferase 1) (p=0.0013). DNMT1 is frequently overexpressed in cancers and drives silencing of tumor suppressor gene via promoter hypermethylation [41] and silencing of differentiation genes and maintenance of a stem-like state [35]. We examined the impact of LATS1 knock-in on TEAD2 transcriptional target DNMT1 (DNA methyltransferase 1)[22], which showed decreased protein expression 3 and 4 days post-LATS1 knock-in (Fig. 3b,f).

As above for individual gene expression, we correlated all TFT geneset enrichments with TTP in recurrent PFA (n=79), which showed TEAD2 was the 65th most negatively correlated TFT geneset enrichment of all 1066 TFTs (Fig. 5f). Kaplan–Meier analysis confirmed that high *TEAD2* target-gene expression enrichment (ssGSEA) was associated with poor survival (n=88) (HR = 2.80; p = 3.6×10^–6^) (Fig. 5g).

### PFA 6q- cells are sensitive to TEAD palmitoylation inhibition and PROTAC-mediated TEAD degradation

We performed dose curve analysis of pan-TEAD palmitoylation inhibitor VT017 [48] in vitro in PFA 6q- cell lines MAF-811 and MAF-928 alongside PFA 6q WT cell line DKFZ-BT232, supratentorial ZFTA::RELA fusion ependymomas EP1NS and 1329 and a panel of comparator paediatric brain tumour cell lines as previously used to further determine PFA 6q- selective effects [9]. IC-50 values of VT107, as measured by ^3^H-thymidine incorporation, showed sensitivity in both PFA 6q- cell lines, showing high nanomolar sensitivity (MAF-811 = 845 nM; MAF-928 = 420 nM) (Fig. 5h). Comparator paediatric brain tumour cell lines showed variable sensitivity to VT107, with IC-50 doses not achieved in a number of cell lines at the broad dose range tested (0.61 nM to 10μM)(Figs. 5h). PFA 6q WT cell line DKFZ-BT232 had a higher IC-50 value (1.36 μM) than PFA 6q- cell lines, although the IC-50 revealed a relatively high sensitivity to the drug compared to non-ependymoma high-grade glioma (HGG) and medulloblastoma (MED) pediatric comparator lines. Collectively, the sensitivity of BT232 and ZFTA::RELA fusion supratentorial ependymoma cell lines in addition to PFA 6q- suggest that ependymomas subtypes are broadly dependent on TEAD activity and implicate dysregulated Hippo signaling that is already known to drive YAP-fusion supratentorial EPN [33].

Proteolysis targeted chimeras (PROTACs) are in clinical trial in the oncology setting and represent a paradigm-shifting therapeutic approach to target transcription factors with several advantages over small molecule inhibitors, with improved efficiency at lower dosing and their ability to overcome drug resistance [46]. These compounds also provide a powerful laboratory tool for selective and rapid knock-down of target proteins. We tested a recently developed pan-TEAD-targeted PROTAC degrader H122 [27] in the PFA 6q- and comparator cell line panel as described above. PFA 6q- demonstrated the lowest IC-50 values for H122 (MAF-811 = 9.8 nM; MAF-928 = 4.5 nM) (Fig. 5i), approximately 100-fold lower than those obtained using VT107. As for VT107, H122 IC-50 s were significantly higher for non-ependymoma HGG and MED cell lines. PFA 6q WT DKFZ-BT232 had a higher IC-50 (25.1 nM) than PFA 6q- cell lines, as did ZFTA::RELA fused supratentorial ependymoma cell lines, as seen using VT107 TEAD inhibition, again suggesting an underlying Hippo pathway dysregulation in EPN as a whole. Mechanistically, H122-treated PFA 6q- cell lines showed complete degradation of TEAD protein after 4 h and endogenous TEAD degradation was not seen up to 24 h post-protein translation inhibition (100μγ/ml cycloheximide) (Fig. 5j), confirming H122 PROTAC on target effects and also demonstrating that PFA 6q- cell lines have an intact ubiquitination degradation pathway that is critical for PROTAC efficacy.

### TEAD2 is enriched in undifferentiated ependymoma cell subpopulations (UEC-A)

Building on our prior work identifying intratumoural heterogeneity in PFA EPN [13, 14, 16], we examined *TEAD2* expression within distinct neoplastic subpopulations. In earlier single-cell analyses, we defined an Undifferentiated Ependymoma Cell type (UEC) population with neural stem-like features and progenitor potential [13].

**Fig. 6:**
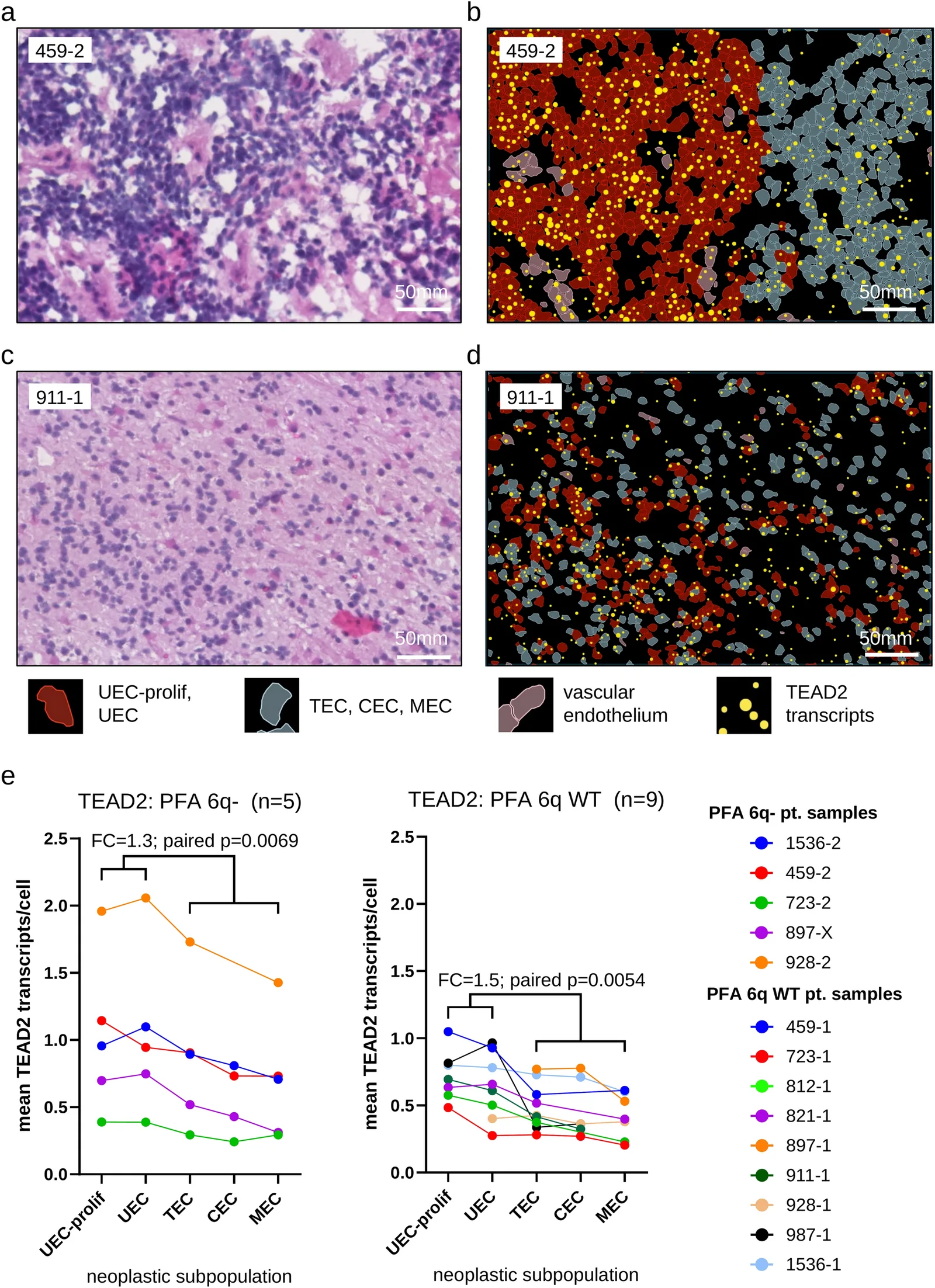
Overexpression of TEAD2 expression in UEC-A PFA stem-like subpopulation. **a** PFA 6q- (sample 459–2) representative H&E staining and **b** corresponding section overlaid colored by UEC-A progenitor cells (red) in relation to more differentiated TEC, CEC and MEC neoplastic subpopulations (grey) and vascular endothelium (mauve) as identified by Xenium in situ spatial transcriptomic analysis, showing differential abundance of TEAD2 transcripts (yellow spots, scaled according to transcript abundance). **c** PFA 6q WT H&E and **d** overlayed cellular subpopulations as described in **b**. **e** Quantification of TEAD2 transcripts in UEC-A cells versus other neoplastic cell types in 5 PFA 6q- and 9 PFA WT samples. Abbreviations: UEC, undifferentiated EPN cell; TEC, transportive EPN cell, CEC, ciliated EPN cell; MEC, mesenchymal EPN cell

Using Xenium in situ spatial transcriptomics, we measured the expression of *TEAD2* and *LATS1* in individual cells in 14 PFA tissue sections (5 PFA 6q- and 9 PFA 6q WT patient samples) (1,096,706 total cells). Subcellular resolution in situ spatial transcriptomics was performed using a panel of 480 custom probes based on previously identified cellular subpopulations in EPN [13–15] and other paediatric brain tumour diagnoses [7, 37, 57] (Supplementary Table 1). Cell segmentation of tissue sections was combined with corresponding custom probe expression to identify cell types using graph-based clustering. Xenium clusters were annotated by comparison of marker genes with EPN single-cell RNAseq (scRNAseq) and Visium spatial transcriptomic neoplastic and non-neoplastic cell subpopulation markers [13, 14] and neoplastic cells were assigned to correspond with major PFA neoplastic subpopulations: undifferentiated EPN cell UEC-A (including a proliferative UEC-A subpopulation (UEC-A prolif.)), ependymally-differentiated transportive TEC and ciliated CEC EPN cells or and mesenchymally-differentiated MEC EPN cells. Significantly higher *TEAD2* expression was seen in UEC and UEC-A proliferative subpopulations than other neoplastic subpopulations (TEC, CEC and MEC) both in PFA 6q- and PFA 6q WT (Fig. 6e). LATS1, was collectively lower in PFA 6q- than PFA 6q WT, but did not show differential expression between UEC and other populations (Supplementary Fig. 1). Gain-of-function activity of TEAD has previously been shown to drive expansion of the neural progenitor pool [6]. Consistent with this, our data indicate that TEADs—particularly TEAD2—underlie UEC-A proliferation, supporting our hypothesis that UEC-A cells represent a proliferative progenitor population contributing to the aggressive behaviour of PFA EPN.

## Discussion

In this study, we show that 6q- is progressively accumulated at successive recurrences and is a prognostically adverse event in PFA EPN. 6q- at any recurrence is associated with shortened survival and accounts for nearly half of deaths. Through analysis of partial versus whole chromosome 6q loss, we demonstrate that *LATS1* deletion underlies this, leading to Hippo pathway dysregulation and downstream activation of TEAD family transcription factors – particularly *TEAD2*. Preclinical testing showed PFA 6q- cell lines were sensitive to TEAD inhibition. Spatial transcriptomics further localised *TEAD2* over-expression to undifferentiated, stem-like UEC-A progenitor cells. Our findings support a model in which chromosome 6q- leads to *LATS1* inactivation, thereby releasing *YAP*, enhancing *TEAD2* transcriptional activity, and this stem-like tumour-cell population. Thus, Hippo pathway dysregulation emerges as a central feature of aggressive, recurrent PFA EPN.

Unlike 1q gain, which appears at presentation and earlier relapses, 6q loss emerges later and increases steadily across recurrences, indicating distinct evolutionary pressures and complementary mechanisms of aggressiveness. This may explain why tumours harbouring both 1q+ and 6q- exhibit the poorest outcomes [5, 15]. Previous research has focused mainly on 1q gain, which mimics p53 suppression through MDM4 overexpression and may be targetable with 5-FU [15]. Our data extend this paradigm by identifying 6q loss as an equally important later driver of rapid progression.

The Hippo pathway has been implicated in many malignancies and regulates cancer hallmarks, many of which have already been implicated in PFA EPN, including angiogenesis, proliferation, epithelial-mesenchymal transition, inflammation and immune evasion [13, 16, 28, 51, 53]. This work aligns with work in colorectal carcinoma, where rare “revival stem cells” emerge in response to cancer therapy and are regulated by Hippo pathway components [12, 49]. Similarly, our data provides initial work to identify TEAD2-high UEC-A cells as potential revival-like progenitors, consistent with prior observations of oncofetal reprogramming [10, 13, 16, 43]. Future studies should aim to further characterise the UEC-A population and improve understanding of its role in high-risk PFA EPN as a potential therapeutic target.

Loss of *LATS1* has previously been implicated as a potential tumorigenic mechanism in supratentorial EPN. Specifically, in the context of the exploration of *YAP1*-fused supratentorial disease. Eder and colleagues described a murine model in which deletion of YAP1's negative regulators LATS1 and LATS2 kinases in NEX-Cre lineage in double conditional knockout mice caused ependymoma-like tumours [11]. This experimental evidence strongly supports our conclusion that Hippo-pathway dysregulation represents a shared oncogenic mechanism in EPN, reinforcing the rationale for developing Hippo-targeted therapies.

Clinically, our findings carry important implications. The rarity of 6q- at PFA presentation means that its significance at that time point, as in the retrospective E-HIT2000 cohort is to be interpreted cautiously [21]. However, all studies on recurrent PFA should be stratified for 6q-. It does appear that PFA ependymoma treated with surgery ± radiation may sometimes be cured or go often years before another relapse while this is not the case in 6q- ependymoma. The ability to restore *LATS1* signalling in vitro suggests that targeting YAP/TEAD interactions or the wider Hippo pathway in 6q- PFA EPN may be feasible. Moreover, the enrichment of *TEAD2* in undifferentiated UEC-A cells raises the possibility of differentiation-based therapeutic strategies.

The current therapeutic landscape for Hippo pathway modulation is encouraging. Approaches include disrupting YAP-TEAD binding, suppressing *TEAD*-regulated transcription, targeting transcriptional machinery, and blocking YAP-TEAD target genes [20]. Yap et al. reported a Phase1/2 trial of the Hippo agent VT3989 in mesothelioma, a high rate of partial responses and stable disease [54]. A Phase 3 trial is planned. VT3989 is an analogue of VT107 that demonstrated PFA 6q- specific anti-proliferative activity in our study. This approach, therefore, offers near-term translational potential for PFA 6q- and possibly YAP1-fused supratentorial disease. Over 40 PROTACs are currently in oncology clinical trials, marked by the recent FDA approval of vepdegestrant for advanced or metastatic breast cancer [26]. The high sensitivity of PFA 6q- cell lines to TEAD PROTAC H122 provide rationale for further preclinical testing of this breakthrough therapeutic strategy. Given the dismal prognosis of relapsed PFA EPN with 6q-, prioritising clinical translation of these findings is essential.

Future research should map the full spectrum of Hippo pathway dysregulation in all subtypes of EPN, including upstream regulators and other YAP/TEAD targets. Pre-clinical 6q-/LATS1 models will be crucial for evaluating candidate inhibitors, but we must simultaneously and urgently consider how we translate this new biological knowledge. However, we are confident that integrating 6q status into prospective clinical trials will refine risk stratification and guide therapy in recurrent disease.

In conclusion, loss of chromosome 6q, and consequent *LATS1* deletion emerge as a key molecular driver of recurrence and poor outcome in PFA EPN, acting through Hippo pathway dysregulation and *TEAD2*-mediated proliferative programs. These insights illuminate a new biological axis of ultra-high-risk ependymoma and provide a rationale for developing Hippo-targeted therapeutic approaches for affected children.

## Key points

(1) Tumour suppressor *LATS1* is in the minimal region of chromosomal loss in PFA 6q-

(2) *LATS1* deletion drives Hippo pathway dysregulation and YAP/TEAD activation.

(3) Restoring *LATS1* expression or inhibiting TEAD suppresses growth in vitro, revealing a targetable pathway.

## Supplementary Information

Below is the link to the electronic supplementary material.

Supplementary file1 (XLSX 23 KB)

Supplementary file2 (TIF 1793 KB)

## Data Availability

Data Availability: The datasets generated and analysed during the current study are available at GEO (Superseries GSE327676). Existing published datasets referenced in this study are available from their respective sources as cited in the manuscript.
